# Chuna Manual Therapy or Electroacupuncture with Pregabalin for Chemotherapy-Induced Peripheral Neuropathy: A Randomized Controlled Pilot Study

**DOI:** 10.3390/jcm13133916

**Published:** 2024-07-04

**Authors:** Yeon-Woo Lee, Ilkyun Lee, Jin-Hyun Lee, Min-Geun Park, Ji-Hoon Kim, Yoon-Young Sunwoo, Man-Suk Hwang, Tae-Yong Park

**Affiliations:** 1 Department of Korean Medicine Rehabilitation, Pusan National University Korean Medicine Hospital, Yangsan 50612, Republic of Korea; lyw2667@gmail.com; 2 School of Korean Medicine, Pusan National University, Yangsan 50612, Republic of Korea; 3 Department of Surgery, Catholic Kwandong University International St. Mary’s Hospital, Incheon 22711, Republic of Korea; iklee68@gmail.com (I.L.); damian11@ish.ac.kr (M.-G.P.); 4 Institute for Integrative Medicine, Catholic Kwandong University International St. Mary’s Hospital, Incheon 22711, Republic of Korea; doolyjinhyun@empal.com; 5 Department of Surgery, Incheon St Mary’s Hospital, Catholic University College of Medicine, Incheon 21431, Republic of Korea; samryong@catholic.ac.kr; 6 Iksoodang Korean Medicine Clinic, Incheon 21425, Republic of Korea; 127501@hanmail.net

**Keywords:** complementary and alternative medicine (CAM), chemotherapy-induced peripheral neuropathy (CIPN), electroacupuncture (EA), Chuna manual therapy (CMT), pregabalin

## Abstract

**Background:** Chemotherapy-induced peripheral neuropathy (CIPN) is one of the most common side effects of chemotherapy, and effective treatments for CIPN are still lacking. For this reason, there is a growing interest in complementary and alternative medicine as a potential source of nonsurgical treatments for CIPN symptoms alongside pregabalin. One such option being explored is Chuna manual therapy (CMT), a traditional Korean manual therapy. **Methods:** This study compares the effectiveness and safety of using only pregabalin (PG) as a conventional method of treating breast and colorectal cancer patients with CIPN symptoms with a combination of both PG and electroacupuncture (EA) or CMT, while also assessing the feasibility of future large-scale clinical studies. Due to the COVID-19 pandemic, only 74 CIPN patients were recruited to this study. Twenty-five were assigned to the PG group, 26 to the PG + EA group, and 22 to the PG + CMT group for a five-week treatment and a four-week follow-up study. **Results:** For the primary outcome, we evaluated the mean differences in Functional Assessment of Cancer Therapy/Gynecologic Oncology Group-Neurotoxicity (FACT/GOG-Ntx) compared to the baseline at week 5 (visit 4). Although we found that the PG + CMT group showed the biggest difference (−16.64 [95% CI: −25.16, −8.11]) compared to the PG group (−8.60 [95% CI: −14.93, −2.27]) and the PG + EA group (−6.73 [95% CI: −12.34, −1.13]), this finding lacked statistical significance (*p* = 0.2075). In terms of safety, two patients in the PG + CMT group reported side effects: one bruise and one headache. **Conclusions:** The low attrition and high adherence rates of all the groups, and the similar rates of side effects among them, support the feasibility of larger-scale follow-up studies.

## 1. Introduction

Chemotherapy is a standard course of treatment administered to 60–75% of diagnosed cancer patients, and it has been proven to be effective in preventing recurrence and improving the survival rates of patients [1]. Some anticancer drugs, however, are known to be neurotoxic and, thus, capable of altering or damaging not just cancerous cells, but also peripheral nerve fibers, causing a wide range of side effects affecting sensory and motor nerves, such as reduced sensations, sensory anomalies, loss of sensations, hyperesthesia, tactile dysfunction, pain, the weakening of muscles, and muscular seizures [2,3], which can have a debilitating effect on patients’ quality of life. Together, these symptoms form what is known as chemotherapy-induced peripheral neuropathy (CIPN). This condition has been reported in 68.1% of patients on chemotherapy, and it is known to persist in 30% of these patients for 6 months or longer [4].

Not only is CIPN a common side effect of chemotherapy, it can also continue for extended periods of time and/or lead to irreversible sequelae. It is therefore not surprising that interest has been growing in ways to mitigate or prevent CIPN [5]. An increasing variety of pharmacological solutions, including gabapentin, pregabalin, duloxetine, calcium/magnesium, vitamin E, glutathione, amifostine, and amitriptyline, are being used to prevent or treat CIPN in cancer patients, but the consistency of the efficacies of these drug regimens is still in dispute [6]. According to the revised American Society of Clinical Oncology (ASCO) guidelines from 2020, no existing pharmacological solutions have been found to be a proven cure to CIPN [7]. The National Institute for Health and Care Excellence guidelines recommend gabapentin and pregabalin for the temporary relief of symptoms [8]. Pregabalin has undergone the most research as a drug treatment for CIPN-associated pain, with clinicians favoring it for its proven efficacy in mitigating pain and its safety [9]. The analgesic effect of the drug, however, can be rather limited, while the drug is also known to cause its own set of side effects, including dizziness and drowsiness [10]. For cancer patients whose CIPN symptoms are so severe that they do not respond to pregabalin or other such drugs, it may be necessary to reduce the dosage of their anticancer drugs or switch them to other anticancer drugs. When the symptoms are severe, chemotherapy may have to be stopped entirely [11].

Because CIPN can significantly compromise the efficacy of chemotherapy and even directly affect the survival of cancer patients, it is crucial to find complementary and alternative medicine (CAM) strategies to target the condition [11,12]. One such alternative is integrative, non-pharmacological intervention involving acupuncture and/or manual therapy [13,14]. The ASCO guidelines suggest acupuncture as a possible alternative for CIPN that is resistant to pharmacological intervention [7]. Electroacupuncture (EA) has been reported to be more effective than conventional acupuncture in significantly lowering the incidence rates of CIPN [15,16,17]. Additionally, manual therapies such as Chuna manual therapy (CMT), a traditional Korean manual therapy, may have the potential to alleviate neuropathic pain, improve sensory abnormalities, and enhance motor function in CIPN patients [18]. Moreover, CMT has been shown to accelerate the regeneration of damaged nerves and modulate the transmission of pain-related neurotransmitters [19,20]. Therefore, EA and manual therapies may offer some potential as adjunctive therapies for effectively aiding CIPN treatment.

The main hypothesis of this study is that combining pregabalin, which is the conventional drug prescribed to treat CIPN, with non-pharmacological integrative therapies (EA and CMT) can generate synergy in CIPN treatment. To test this hypothesis, a preliminary clinical study was designed as follows. Patients experiencing CIPN symptoms (i.e., colorectal cancer patients on oxaliplatin chemotherapy and/or breast cancer patients on taxanes) were to be randomly assigned to three groups: a control group treated with pregabalin only (PG), a group using pregabalin concurrently with EA (PG + EA), and a group using pregabalin concurrently with CMT (PG + CMT). The purpose was to validate the hypothesis and assess the feasibility of larger-scale future clinical trials, while also generating basic data necessary for developing the protocols for such larger-scale trials.

## 2. Materials and Methods

### 2.1. Study Design

**Figure 1:**
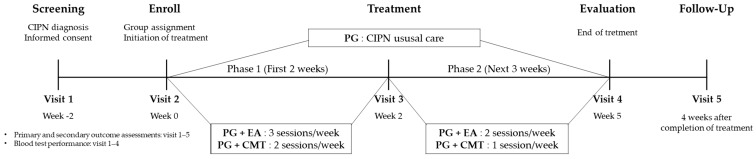
Study schematic diagram. CMT: Chuna manual therapy; EA: electroacupuncture; PG: pregabalin.

This preliminary clinical study took place as a parallel, assessor-blinded, randomized control trial at Catholic Kwandong University International St. Mary’s Hospital in South Korea. The study design was approved by the Institutional Review Board (IRB) in August 2018 (review case no. IS18ENSI0054) and registered with the Clinical Research Information Service (registration no. KCT0004217; https://cris.nih.go.kr/cris/en/search/search_result_st01.jsp?seq=12752, Last Updated Date: 20 August 2019). Figure 1 shows that study schematic diagram.

### 2.2. Participants

(1) Inclusion Criteria

This study targeted patients between the ages of 18 and 85 who either had colorectal cancer and were being treated with oxaliplatin or had breast cancer and were on taxane-based chemotherapy, who were suffering from symptoms of CIPN for at least 1 month, and whose CIPN symptoms were at least Grade-2 level or higher in severity according to the Common Terminology Criteria for Adverse Events (CTCAE) V5.0 (Table S1). Of these patients, those who were not experiencing major disruptions in their daily functions, whose symptoms ranged from Grade 0 to Grade 2 level according to the Eastern Cooperative Oncology Group (ECOG) standards (Table S2), who were capable of reading and answering the questionnaire regarding symptoms on their own, and who gave their consent to participate in the study were selected to participate in the trial.

(2) Exclusion Criteria

The trial excluded patients who had symptoms of neuropathy due to causes other than chemotherapy (e.g., diabetes, peripheral vascular diseases, alcohol use, drug use, or other neurological dysfunctions); who were experiencing or had experienced renal diseases in the past and whose attending physicians thus ruled them out from possible pregabalin prescriptions; who had dermatological legions, fractures, or other such conditions that made it difficult to administer EA, CMT or both; who had severe fear of or resistance toward EA, CMT, or both; who had sought and obtained traditional Korean medical therapies for their CIPN symptoms within the week preceding the start of the trial; who had other medical conditions or factors that made it unsuitable for them to participate in a clinical trial; and who were otherwise deemed by their attending physicians as unfit to participate. Pregnant women and breastfeeding mothers were also excluded.

### 2.3. Randomization and Blinding

Patients who had given written consent to participate in the trial were screened for demographic information, medical history, and medication records; submitted to neurological examinations and laboratory tests; had vital signs measured; and completed questionnaires before they were finally registered as subjects in the trial. The registered patients were then given a run-in period of seven days, during which they suspended their existing drug treatment regimes so that possible interactions with those drugs could be ruled out (Table S3). The patients were then randomly assigned and block-stratified to the three groups, with 25 patients in the PG group, 26 in the PG + EA group, and 22 in the PG + CMT group. A randomization table and envelopes were created by an independent professional statistician and an individual who was not involved in the study, respectively. After obtaining informed consent, screening numbers were assigned according to the order of the patients’ visits. The randomization envelopes were then distributed according to the order of assignment of the patients assigned to one of the three groups. Blinding of the researchers and participants was not possible owing to the nature of the EA or CMT treatment intervention. Thus, independent researchers who did not participate in the treatment evaluated the outcomes while being blinded to prevent bias.

### 2.4. Intervention

#### 2.4.1. PG Group

Each patient in the PG group was administered Lyrica 75 mg capsules (pregabalin, Pfizer, New York, NY, USA) twice a day for a total of 150 mg per day. Where necessary, their physicians could increase the pregabalin dose to 300 mg (150 mg per capsule twice) per day. Changes made to the participants’ dosages were specified in the symptom record sheet.

#### 2.4.2. PG + EA Group

**Figure 2:**
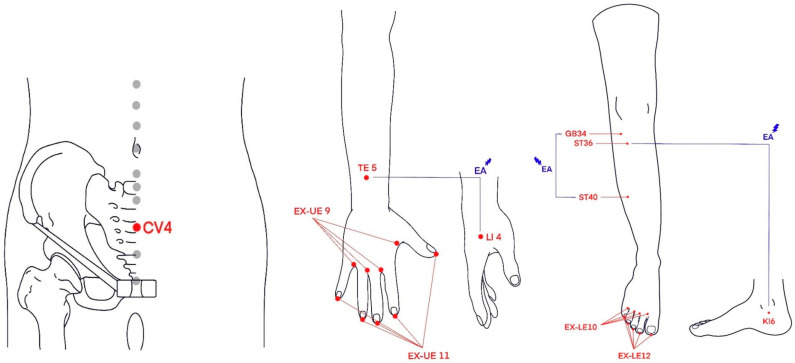
Acupoints combination: (ST 40, GB 34), (EX-LE 10, EX-UE 11), (TE 5, LI 4), (ST 36, KI 6), CV 4, and (EX-LE 12, EX-UE 9).

Patients in the PG + EA group were administered pregabalin in the same schedule and dosage as those in the PG group. These patients were additionally given three EA sessions per week for the first 2 weeks of the trial, after which the frequency of their sessions was lowered to twice per week for the remaining 3 weeks. The different frequencies of EA sessions were intended implemented to explore the optimal treatment frequency for a larger-scale clinical study. The acupuncture points targeted for the EA were selected through a systematic process. Twenty-four acupuncture points frequently used in 25 RCTs on acupuncture for CIPN were identified. These points were then subjected to network analysis using the Jaccard similarity coefficient to identify highly similar combinations [21]. The final selection of acupoints was further refined through an expert Delphi process to ensure clinical relevance and consensus. The final list of EA points was decided in consultation with experts. Electric needles were administered to ST 40, GB 34, TE 5, LI 4, ST 36, and KI 6, while no electric simulation was provided to EX-LE10, EX-UE 11, or CV 4 (Figure 2). Where deemed necessary by the attending acupuncturist, depending on the locations of CIPN symptoms, additional electric stimulation could be provided to EX-LE 12 and EX-UE 9. Although EA was to be performed on acupuncture points on both sides of the body in principle, breast cancer patients who were experiencing edema in their upper body after surgery were spared EA on the points in the affected areas of their body. For EA, disposable stainless-steel needles (spring handle needle, 0.2 mm × 30 mm; Dongbang Medical Co., Ltd., Seongnam, Republic of Korea) were inserted about 1 cm below the surface of the skin and left in the skin for 20 min. For the points targeted for electric stimulation, an EA machine (STN-330 with a maximum output frequency of 250 Hz, output currents of low 7.3 mA and high 13.0 mA; StraTek Company Limited, Anyang, Republic of Korea) was used to generate 2 Hz mixed pulses for 20 min (see Table S4 for details).

#### 2.4.3. PG + CMT Group

Patients in the PG + CMT group were likewise treated with pregabalin in the same manner and dosage. In addition, they were given two CMT sessions per week for the first 2 weeks of the trial and then one CMT session per week for the remaining 3 weeks. This difference in frequency was also used to determine the appropriate frequency of CMT to be administered to patients in a future larger-scale study. The colorectal and breast cancer patients participating in this trial were given a type of CMT known as Sunwoo manual therapy (SMT). SMT, a type of CMT technique, may be effective in managing neuropathic conditions due to its ability to modulate the autonomic nervous system and regulate internal organ functions by mobilizing joints and enhancing circulation [22]. This therapy had the patient lying on the edge of a bed in a lateral position, with the bed-touching leg kept straight on the bed while the other leg hovered out of the bed. The therapist would then hold the patient by the shoulder and use the patient’s weight to mobilize their joints, lasting approximately 4–5 min on each side, for a total of 8–10 min on both sides per set. Three sets were performed for a total duration of 30 min. Breast cancer patients whose upper body the therapist could not and should not apply consistent pressure were instead given CMT for the meridian sinew system and nerve relaxation, as judged fitting by the clinician.

## 3. Outcome Measurements

Given the lack of established objective diagnostic criteria for CIPN, this study relied on validated patient-reported outcome measures to assess the multidimensional aspects of quality of life (QoL) in CIPN patients [23]. Three questionnaires were employed: Functional Assessment of Cancer Therapy/Gynecologic Oncology Group-Neurotoxicity (FACT/GOG-Ntx, version 4) [24], European Organization for Research and Treatment of Cancer Quality of Life Questionnaire CIPN20 (EORTC QLQ-CIPN20) [25], and European Organization for Research and Treatment of Cancer Quality of Life Questionnaire Core 30 (EORTC QLQ-C30, version 3) [26].

### 3.1. Primary Outcome

The primary outcome, a change in the QoL of CIPN patients, was measured as the mean difference of the FACT/GOG-Ntx scores [27] between visit 2 (baseline) and visit 4 (week 5). The FACT/GOG-Ntx questionnaire provides a comprehensive assessment of QoL in cancer patients, including a neurotoxicity subscale specifically designed to evaluate CIPN-related symptoms and their impact on daily life. This questionnaire calculates the total score by summing the points from five subscales points: physical, social/family, emotional, functional, and neurotoxicity domains. Higher total scores indicate better QoL [19].

### 3.2. Secondary Outcomes

For the secondary outcomes, the mean differences and the rates of difference in the FACT/GOG-Ntx scores were checked on visit 3 (week 2) and visit 5 (the follow-up), and compared to the scores measured on visit 2 (baseline) and visit 4 (week 5). To determine CIPN severity, the EORTC QLQ-CIPN20 assessed impacts across sensory, motor, autonomic functions, and daily living activities. The mean differences and rates of differences in the EORTC QLQ-CIPN 20 scores were measured at visit 3 (week 2), visit 4 (week 5), and visit 5 (the follow-up) and compared to visit 2 (baseline). The total score is calculated by summing the scores of each item, with higher scores indicating worse CIPN-related QoL [28]. Finally, to evaluate CIPN patient QoL, EORTC QLQ-C30 scores were checked on visit 3 (week 2), visit 4 (week 5), and visit 5 (the follow-up) and compared to their scores on visit 2 (baseline) [29]. It consists of 30 items assessing physical, emotional, social functioning, symptom experience, and overall health status. The total score is calculated by summing the scores of items 1 to 28, with higher scores indicating better QoL [30].

### 3.3. Exploratory Efficacy

The chemotherapy completion rate was measured for patients in each group who completed their scheduled chemotherapy. Five patients were selected at random from each group to undergo nerve conduction study (NCS) at visit 2 (baseline), visit 3 (week 2), and visit 5 (the follow-up) to determine any objective and quantifiable changes in their symptoms. All NCS results were interpreted by a rehabilitation medicine specialist with over five years of experience, particularly in identifying abnormalities and correlating them with clinical symptoms of neuropathy.

### 3.4. Safety and Adverse Event Monitoring

The participating patients were given physical examinations and checked for their vital signs as well as subjective and objective symptoms at each visit to determine whether they had experienced any adverse events. Patients who reported or displayed adverse events were subjected to follow-up monitoring to determine the causes of these events. The IRB was to be informed immediately of any patients who were to be removed from the trial due to having experienced major adverse events.

### 3.5. Statistical Analysis

Statistical analysis was mainly performed using the full analysis set (FAS). All statistical analyses were given two-sided tests, with a significance level of five percent. A mixed-effects model for repeated measures was set up and used to verify the mean differences in the FACT/GOG-Ntx scores measured on the primary assessment and the differences between groups, as reported on the secondary assessment. Exploratory efficacy testing involved either an ANOVA or a Kruskal–Wallis test in relation to continuous variables and Pearson’s chi-square test or Fisher’s exact test in terms of categorical variables. The safety assessment involved analyzing possible correlations among the frequency of adverse events, the number of affected patients, severity, and treatment using descriptive statistics for each group, with additional chi-square or Fisher’s exact tests performed when necessary.

## 4. Results

**Figure 3:**
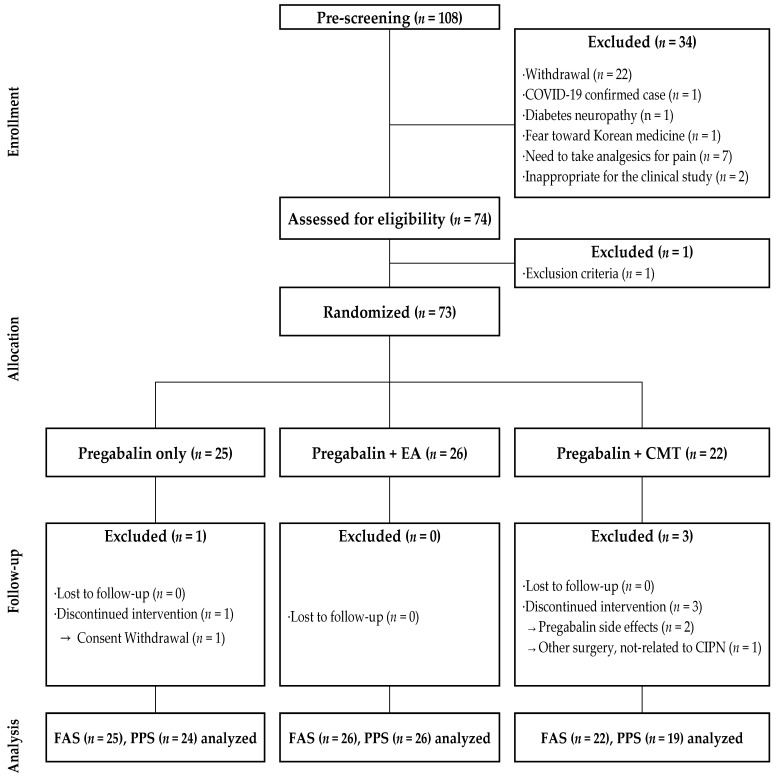
Flowchart of the clinical trial. CMT: Chuna manual therapy; EA: electroacupuncture; FAS: full analysis set; PPS: per-protocol set.

Although the initial plan for this study was to register a total of 90 patients (30 for each group), the COVID-19 pandemic and related delays led to the eventual registration of only 74 patients. Of these, 25 were assigned to the PG group, 26 to the PG + EA group, and 22 to the PG + CMT group. After screening, one patient was found to meet part of the exclusion criteria and was therefore removed from the trial, leaving 73 patients randomly assigned to the groups. The follow-up led to the removal of two more patients due to pregabalin-related side effects, one patient due to non-CIPN-related disk herniation, and one patient after rescinding their consent to the study. Therefore, 69 patients managed to continue to the end of the trial (Figure 3). Table 1 provides a summary of the demographics of the participating patients. No statistically significant differences were observed in relation to variables such as age, height, or BMI.

### 4.1. Primary Outcome

**Figure 4:**
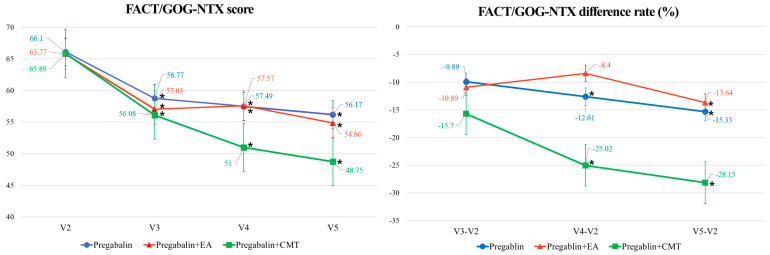
Line graph of outcomes according to baseline and treatment periods. CMT: Chuna manual therapy; EA: electroacupuncture; FACT/GOG-Ntx: Functional Assessment of Cancer Therapy/Gynecologic Oncology Group-Neurotoxicity; V: visit; V1: screening; V2: baseline; V3: treatment 2 weeks; V4: treatment 5 weeks; V5: post-follow-up. *: significant difference within group analysis from baseline as *p* < 0.05.

The mean differences in the FACT/GOG-Ntx scores between visit 2 (baseline) and visit 4 (week 5) were −8.60 (95% CI: −14.93, −2.27), −6.73 (95% CI: −12.34, −1.13), and −16.64 (95% CI: −25.16, −8.11) in the PG, PG + EA, and PG + CMT groups, respectively (Table 2). Within each group, the FACT/GOG-Ntx scores demonstrated a statistically significant decrease, indicating a significant treatment effect (all *p* < 0.05). The greatest decrease in FACT/GOG-Ntx scores was observed in the PG + CMT group, although no statistically significant differences were found between the groups (*p* = 0.2075) (Table 2, Figure 4).

### 4.2. Secondary Outcomes

The rates of differences in FACT/GOG-Ntx scores between visit 2 (baseline) and visit 4 (week 5) were −12.61 (95% CI: −23.78, −1.43), −8.40 (95% CI: −17.20, 0.40), and −25.02 (95% CI −38.20, −11.83) in the PG, PG + EA, and PG + CMT groups, respectively (Table 2). Intragroup comparisons suggested observable treatment effects in the PG and PG + CMT groups (*p* < 0.05), though these differences were observed within the PG + EA group. No statistically significant differences emerged between groups (*p* = 0.0738). The FACT/GOG-Ntx score of the PG + EA group, in fact, increased during the trial, contrary to the cases of the PG and PG + CMT groups (Figure 4). The mean differences and the rates of differences between visit 2 (baseline) and visit 3 (week 2) were −7.32 (95% CI: −13.77, −0.87) and −9.89 (95% CI: −21.35, 1.58) for the PG group, −7.31 (95% CI: −13.09, −1.53) and −10.89 (95% CI: −22.08, 0.30) for the PG + EA group, and −11.50 (95% CI: −19.62, −3.38) and −15.70 (95% CI: −26.86, −4.53) for the PG + CMT group. No statistical significance was observed in either the mean differences (*p* = 0.7926) or the rates of differences (*p* = 0.7019) (Table 2). The mean differences and the rates of differences in the FACT/GOG-Ntx scores between visit 2 (baseline) and visit 5 (the follow-up) were −9.92 (95% CI: −15.77, −4.07) and −15.33 (95% CI: −25.23, −5.44) for the PG group, −9.58 (95% CI: −15.31, −3.85) and −13.64 (95% CI: −22.37, −4.91) for the PG + EA group, and −18.73 (95% CI: −26.59, −10.87) and −28.15 (95% CI: −40.62, −15.69) for the PG + CMT group—once again, with no statistical significance in either the mean differences (*p* = 0.1737) or the rates of differences (*p* = 0.1112). Between visit 3 (V3) and visit 4 (V4), the rate of difference in the FACT/GOG-Ntx scores increased only for the PG + EA group (Table 2, Figure 4). The EORTC QLQ-CIPN 20 and EORTC QLQ C30 scores also indicated that no statistically significant differences could be observed between the groups in either the mean differences or the rates of differences between visit 2 (baseline) and visit 3 (week 2), visit 4 (week 5), or visit 5 (the follow-up) (Table 2).

### 4.3. Exploratory Efficacy

The exploratory efficacy of the trial was also measured by additionally verifying the chemotherapy completion rate and performing NCS. The former was measured by verifying the actual percentage of patients who had completed all their scheduled chemotherapy sessions. No statistically significant differences emerged between the groups (*p* = 0.5752) (Table 3). NCS was performed on 15 patients, all of whom were randomly selected from the groups. Five of these patients reported CIPN symptoms, even though their NCS results were normal. The remaining 10 patients showed adverse NCS results and CIPN symptoms. Four of the patients showed improvement in their CIPN symptoms, even though their NCS results remained abnormal. All four patients came from the PG + EA and PG-CMT groups. There were also six patients whose NCS results were abnormal and whose CIPN symptoms did not abate.

### 4.4. Safety and Adverse Event Monitoring

Of the 73 patients who had initially been registered with the trial, 17 reported adverse events, including three (12%) in the PG group, five (19.23%) in the PG + EA group, and nine (40.61%) in the PG + CMT group. The differences in the number of adverse events reported between the groups lacked statistical significance (*p* = 0.0538) (Table 4). One serious adverse event was reported, which involved a patient in the PG + CMT group who was hospitalized due to an acute herniated disk in the lower back. Although the event was Level 3 serious (i.e., requiring hospitalization or prolongation of existing hospitalization), it bore no direct relation to the intervention performed on the patient. The causality analysis revealed that none of the three cases of adverse events reported by the PG group were related to pregabalin. Likewise, the five cases of adverse events from the PG + EA group were not likely to be related (one) or not at all related (four) to the intervention in concern (Table S5). Of the nine cases of adverse events from the PG + CMT group, two involved bruises and headaches that were definitely related to the therapy in question, but these were nonserious events from which the patients recovered in a short time. Of the remaining seven, one was judged as not likely to be related, and the other six were deemed to be unrelated to CMT (Table S5).

## 5. Discussion

This preliminary clinical study is a well-designed, randomized, and comparative study for determining how efficacious and safe nonpharmacological interventions such as acupuncture and CMT can be when concurrently administered alongside the typical drugs used to treat CIPN symptoms. The objective of this study was to test the feasibility of larger-scale future clinical trials and to help explore a possible design for such studies. Although two patients in the PG + CMT group left the trial in the middle of the process due to pregabalin-related headaches, no patients dropped out of the trial due to side effects from either EA or CMT (Table S6). Notably, both EA and CMT demonstrated high patient adherence and low attrition rates. The lack of major side effects, low turnover rates, and high conformity rates served to minimize possible distortions in the study results and enhance the consistency and validity of the results of this study, providing preliminary evidence on the potential feasibility of larger-scale future clinical trials.

This preliminary study also produced the following implications for future clinical trials. First, despite its small sample size (*n* = 73), this pilot study offers valuable preliminary evidence on the potential benefits of EA and CMT as adjunctive therapies for CIPN. The results, particularly the notable improvement in neurological symptoms and QoL observed in the PG + CMT group, warrant further investigation in a larger-scale trial. The high adherence and low attrition rates across all groups underscore the feasibility of conducting such a trial. Moreover, future research with a larger sample size is warranted to definitively assess the efficacy and clinical significance of these non-pharmacological interventions in CIPN management.

Second, three EA sessions per week and two CMT sessions per week are recommended for future trials. In this preliminary study, patients were treated with three EA sessions or two CMT sessions per week for the first two weeks and were then given two EA sessions or one CMT session per week for the remaining three weeks. Despite the absence of a washout period or crossover clinical trial design in our study, which made it challenging to evaluate the appropriateness of the three times per week and two times per week treatment periods, we observed a trend towards greater improvement with the three times per week treatment. Given these findings, despite the limited evidence, future studies should investigate the optimal treatment frequency for CIPN, potentially initiating with three sessions per week for both EA and CMT. Figure 4 illustrates a greater degree of symptom improvement during the initial two weeks compared to the last three weeks for both the PG + EA and PG + CMT groups. Specifically, while the difference in the rate of amelioration between the first 2 weeks and the last 3 weeks of the trial was marginal for the PG + CMT group, symptoms actually worsened in patients in the PG + EA group in the last 3 weeks compared to the first 2 weeks, likely due to the changed frequency of the EA treatments. These findings suggest that a higher frequency of EA and CMT may be associated with greater therapeutic benefits for CIPN. Further research is needed to investigate the optimal frequency, potentially initiating with three sessions per week and incorporating a washout period to mitigate carryover effects.

Third, NCS may not be necessary for future trials, as the NCS performed as part of monitoring exploratory efficacy for this preliminary study failed to capture changes in patients’ conditions with sufficient levels of objectivity. However, the chemotherapy completion rate may still retain value as a supplementary indicator, as it reflects both the primary and secondary outcomes. It should also be noted that most patients felt overburdened about the fact that they had to fill out both the EORTC-QLQ-CIPN 20 and EORTC-QLQ-C30 questionnaires, while the results obtained from analyzing the answers to both questionnaires were hardly different. Therefore, only one of the questionnaires may be used in future trials.

Fourth, any future trial would require 285 subjects in total, divided into three groups of 95 each. The original design for this study settled on a minimum number of subjects needed to test the efficacy and safety of nonpharmacological, traditional medicine–based interventions based on existing clinical studies similarly targeting CIPN patients [15,17,31]. The number of subjects needed for the larger trial was then determined based on the analysis of the differences in each group’s FACT/GOG-Ntx scores. In future trials, each group will require at least 76 subjects to satisfy the requirements of a 5% significance level and an 80% power. Given the fact that 20% of patients left this preliminary study’s trial midway, a minimum of 95 patients would be necessary for each group, for a total of 285 in future trials. The larger the sample size, the greater the statistical significance and external validity of the results. Additionally, the reliability of the results will also be improved, as larger sample sizes allow for the identification of the sizes of small effects and the discovery of rare side effects.

This study is significant for its objective and evidence-based selection of acupuncture points to test the efficacy of EA. Most clinical studies on acupuncture have so far been conducted with acupuncture points that were generally chosen on the basis of textbooks or expert consensus, without detailed evidence. For this study, however, we chose acupuncture points by sampling them from quality randomized clinical studies and subjecting them to a network analysis and final screening by experts [21]. This systematic approach was used to provide a more objective and scientific basis for clinically studying and demonstrating the efficacy of acupuncture. Furthermore, this study is among the pioneering clinical studies in South Korea on integrative medicine for CIPN. Although Koreans are accustomed to taking an integrative approach rooted in both modern and traditional medicines for a variety of diseases [32,33], there is a significant dearth of studies specifically aimed at CIPN. Kim et al. (2013) sought to establish research protocols for CIPN [31], but the protocols they proposed were limited to the comparison of real acupuncture versus sham acupuncture. There is another study on the effectiveness of EA intervention for breast cancer patients with CIPN symptoms, but it was conducted and published overseas [34]. To our knowledge, this preliminary study is among the first to attempt to analyze and demonstrate the efficacy of integrative medicine in treating CIPN symptoms in Korean patients, and it therefore carries clinical significance.

CMT has shown potential in managing various conditions, including pain, sensory abnormalities, and motor dysfunction, and it has been suggested to promote nerve regeneration and modulate pain transmission [18,19,20]. Given these potential benefits, CMT was included in this study as a complementary therapy for CIPN, alongside EA. This study is also among the first well-designed clinical studies in Korea to provide evidence of the effectiveness of CMT in treating chemotherapy-related side effects such as CIPN. Although the results did not demonstrate a statistically significance difference in efficacy between the CMT group and the other groups, this study suggests that CMT may have positive effects for patients, warranting further research on its application in CIPN treatment.

Although this study did not directly analyze the minimal clinically important difference (MCID) of the FACT/GOG-Ntx questionnaire, we assessed the clinical significance by referring to the MCID range (1.38–3.68) reported in previous studies for each subscale [35]. The MCID values for each intervention in the primary outcome—the change in the total FACT/GOG-Ntx score—were 4.42 for PG, 5.49 for PG + EA, and 6.03 for PG + CMT, all exceeding the clinically valid MCID values (Table S6). This suggests that each intervention may have a clinically meaningful effect on patients’ QoL. Although there is no research on MCID values for between-group comparisons, the PG + CMT group showed a 1.61-point greater improvement in the primary outcome compared to the PG group. This may be a clinically meaningful difference, considering the MCID range within groups reported in previous studies (Table S6). Nevertheless, given the lack of research on MCID between-group comparisons, careful interpretation is required. Although not statistically significant in this study, differences exceeding the MCID were observed in some between-group comparisons, suggesting the possibility of obtaining significant results if clinical research is conducted with a sufficient sample size.

Moreover, this study is limited by the potential bias due to the inherent lack of blinding in CAM therapies and the reliance on subjective assessments due to the absence of established objective diagnostic criteria for CIPN [36]. However, we mitigated potential bias through rigorous assessor blinding and employed diverse assessment tools to evaluate the multidimensional aspects of CIPN and treatment efficacy comprehensively. Additionally, the COVID-19 pandemic posed challenges in patient recruitment, limiting our ability to reach the target sample size of 90 participants. Nevertheless, this study successfully determined appropriate frequencies for EA and CMT interventions in CIPN patients, paving the way for larger, multi-institutional clinical trials to verify the efficacy of combined therapies (PG + EA and PG + CMT). Such trials would provide higher quality evidence on the efficacy of an integrative medicine approach for CIPN management.

## Figures and Tables

**Table 1:** Demographic characteristics *p*-values (ANOVA analysis).

	PG (*n* = 25)	PG + EA (*n* = 26)	PG + CMT (*n* = 22)	*p*-Value
Age (mean, SD; years)	61.6, 10.3	54.3, 9.6	56.6, 12.6	0.0543
Sex (*n*, %) ^‡^				
Male	6 (24.00)	7 (26.92)	2 (9.09)	0.2727
Female	19 (76.00)	19 (73.08)	20 (90.91)	
Height (mean, SD; cm)	159.2, 6.6	160.5, 7.4	157.3, 5.8	0.2507
Weight (mean, SD; kg)	62.7, 12.0	62.6, 10.3	59.1, 7.3	0.3938
BMI (mean, SD; kg/m^2^)	24.7, 3.8	24.4, 4.0	23.9, 2.9	0.7947
SBP (mean, SD; mmHg)	121.4, 13.6	119.4, 18.5	121.9, 20.3	0.8762
DBP (mean, SD; mmHg)	71.2, 10.4	73.2, 12.7	75.3, 7.3	0.4279
Type of cancer (*n*, %) ^‡^				
Colorectal cancer	11 (44.00)	11 (42.31)	9 (40.91)	0.977
Brest cancer	14 (56.00)	15 (57.69)	13 (59.09)	

CMT: Chuna manual therapy; EA: electroacupuncture; DBP: diastolic blood pressure; m: mean; SBP: systolic blood pressure; SD: standard deviation; PG: pregabalin. ^‡^: χ^2^ test.

**Table 2:** Comparison of mean differences and difference rates of primary and secondary outcomes (FAS analysis).

Mean Difference						Difference Rate (%)			
	Visit	PG (*n* = 25)	PG + EA (*n* = 26)	PG + CMT (*n* = 22)	*p*-Value	PG (*n* = 25)	PG + EA (*n* = 26)	PG + CMT (*n* = 22)	*p*-Value
FACT/GOG-Ntx score	V3–V2	−7.32 (−13.77, −0.87) *	−7.31 (−13.09, −1.53) *	−11.50 (−19.62, −3.38) *	0.7926	−9.89 (−21.35, 1.58)	−10.89 (−22.08, 0.30)	−15.70 (−26.86, −4.53) *	0.7019
	V4–V2	−8.60 (−14.93, −2.27) ^¶,^*	−6.73 (−12.34, −1.13) ^¶,^*	−16.64 (−25.16, −8.11) ^¶,^*	0.2075	−12.61 (−23.78, −1.43) *	−8.40 (−17.20, 0.40)	−25.02 (−38.20, −11.83) *	0.0738
	V5–V2	−9.92 (−15.77, −4.07) *	−9.58 (−15.31, −3.85) *	−18.73 (−26.59, −10.87) *	0.1737	−15.33 (−25.23, −5.44) *	−13.64 (−22.37, −4.91) *	−28.15 (−40.62, −15.69) *	0.1112
EORTC QLQ-CIPN 20	V3–V2	−4.16 (−7.83, −0.49) *	−5.00 (−7.96, −2.04) *	−6.50 (−11.68, −1.32) *	0.5673	−11.92 (−22.90, −0.95) *	−12.00 (−18.75, −5.24) *	−17.28 (−32.77, −1.79) *	0.8038
	V4–V2	−5.12 (−8.69, −1.55) *	−7.00 (−9.51, −4.49) *	−7.64 (−13.07, −2.20) *	0.5668	−14.32 (−24.86, −3.79) *	−18.08 (−23.35, −12.81) *	−19.01 (−34.43, −3.59) *	0.4870
	V5–V2	−5.80 (−9.25, −2.35) *	−7.42 (−10.42, −4.43) *	−7.77 (−13.65, −1.90) *	0.6945	−15.89 (−26.42, −5.36) *	−19.09 (−25.63, −12.55) *	−17.74 (−35.33, −0.15) *	0.2630
EORTC QLQ-C30	V3–V2	1.19 (−1.59, 3.96)	2.38 (−0.32, 5.09)	1.67 (−1.30, 4.64)	0.7994	2.78 (−4.96, 10.51)	6.92 (−0.67, 14.51)	8.50 (0.03, 16.97) *	0.5855
	V4–V2	0.05 (−2.73, 2.83)	2.95 (0.28, 5.61) *	0.21 (−2.82, 3.24)	0.1970	0.23 (−7.50, 7.97)	7.50 (0.07, 14.94) *	5.66 (−3.03, 14.35)	0.3900
	V5–V2	0.18 (−2.60, 2.95)	1.33 (−1.34, 3.99)	1.46 (−1.57, 4.49)	0.7491	1.19 (−6.54, 8.93)	4.28 (−3.15, 11.71)	8.06 (−0.64, 16.75)	0.5088

CMT: Chuna manual therapy; EA: electroacupuncture; PG: pregabalin; V: visit; V1: screening; V2: baseline; V3: treatment 2 weeks; V4: treatment 5 weeks; V5: post follow-up. ^¶^: primary outcome; *: significant difference within group analysis from baseline as *p* < 0.05. Least squares mean difference and *p*-values were analyzed by mixed model for repeated measures (MMRM) with the baseline scores, groups, cancer types, and visits as fixed factors, including group × visit and baseline × visit (satisfying normality and homoscedasticity).

**Table 3:** Exploratory efficacy parameters: Anticancer completion.

	PG (*n* = 5)	PG + EA (*n* = 3)	PG + CMT (*n* = 3)	*p*-Value
Anticancer completion rate (%)	5.56 (0, 10)	16.67 (0, 20)	7.14 (5.56, 11.11)	0.5752

CMT: Chuna manual therapy; EA: electroacupuncture; PG: pregabalin.

**Table 4:** Adverse events (safety set analysis).

	PG (*n* = 25)	PG + EA (*n* = 26)	PG + CMT (*n* = 22)	*p*-Value
Adverse events (*n*, %) ^‡^				
Yes	3 (12.00)	5 (19.23)	9 (40.91)	0.0538
No	22 (88.00)	21 (80.77)	13 (59.09)	

CMT: Chuna manual therapy; EA: electroacupuncture; PG: pregabalin. ^‡^: Chi-square test.

## Data Availability

Data are available on request due to privacy/ethical restrictions.

## Supplementary Materials

The following supporting information can be downloaded at: https://www.mdpi.com/article/10.3390/jcm13133916/s1, Table S1: CIPN symptoms (CTCAE v5.0); Table S2: ECOG performance status scale; Table S3: Clinical trial schedule; Table S4: Checklist for STRICTA (STandards for Reporting Interventions in Clinical Trials of Acupuncture) 2010; Table S5: Adverse event details; Table S6. Comparison of mean differences in NTX subscales of primary and secondary outcomes (FAS analysis). References [37,38] are cited in the Supplementary Materials.
