# Supplementary material for: Chuna Manual Therapy or Electroacupuncture with Pregabalin for Chemotherapy-Induced Peripheral Neuropathy: A Randomized Controlled Pilot Study
Source: J Clin Med. 2024 Jul 4;13(13):3916. doi: 10.3390/jcm13133916 (PMC11242039; doi:10.3390/jcm13133916)
Supplement: Supplementary file 1 [file jcm-13-03916-s001.zip › jcm-2996451-supplementary.pdf]

Table S1. **CIPN symptoms (CTCAE v5.0)**

Nervous system disorder					
**CTCAE Term**	**Grade 1**	**Grade 2**	**Grade 3**	**Grade 4**	**Grade 5**
Peripheral motor neuropathy	Asymptomatic; clinical or diagnostic observations only	Moderate symptoms; limiting instrumental ADL	Severe symptoms; limiting self-care ADL	Life-threatening consequences; urgent intervention indicated	Death
Peripheral sensory neuropathy	Asymptomatic	Moderate symptoms; limiting instrumental ADL	Severe symptoms; limiting self-care ADL	Life-threatening consequences; urgent intervention indicated	-

NIH. National cancer institute. Division of cancer treatment & diagnosis (DCTD). Common Terminology Criteria for Adverse Events (CTCAE). Version 5.0. November 27, 2017. https://ctep.cancer.gov/protocolDevelopment/electronic_applications/docs/CTCAE_v5_Quick_Reference_5x7.pdf

Table S2. ECOG performance status scale

GRADE	ECOG PERFORMANCE STATUS
0	Fully active, able to carry on all pre-disease performance without restriction
1	estricted in physically strenuous activity but ambulatory and able to carry out work of a light or sedentary nature, e.g., light house work, office work
2	Ambulatory and capable of all selfcare but unable to carry out any work activities; up and about more than 50% of waking hours
3	Capable of only limited selfcare; confined to bed or chair more than 50% of waking hours
4	Completely disabled; cannot carry on any selfcare; totally confined to bed or chair
5	Dead

Oken MM, Creech RH, Tormey DC, Horton J, Davis TE, McFadden ET, Carbone PP. Toxicity and response criteria of the Eastern Cooperative Oncology Group (ECOG). Am J Clin Oncol. 1982 Dec;5(6):649-655. PMID: 7165009.

Table S3. Clinical trial schedule

Period	Screening	No treatment run-in period	Baseline	Treatment				Follow-up	Early termination
**Clinical trial schedule**	**Visit 1**		**Visit 2**		**Visit 3**		**Visit 4**	**Visit 5**	
**Day**	**-14 ~ -8**	**-7 ~ -1**	**1**	**1 ~ 14**	**14 ± 3**	**14 ~ 35**	**35 ± 3**	**63 ± 3**	
Informed consent	O								
Demographic information	O								
Medical history	O		O						
Medication records	O		O		O		O	O	O
Vital signs	O		O		O		O	O	O
BMI, body composition test	O		O		O		O	O	
Chemotherapy plan information	O								
Laboratory test	O								
Neurological exam	O						O		O
Eligibility assessment	O		O						
Randomization			O						
PG			O		O				
PG + EA			O (3 times/wk)		O (2 times/wk)		O		
PG + CMT			O (2 times/wk)		O (1 time/wk)		O		
Standard safety lab			O		O		O		
hCG	O		O		O		O		
ECG	O								
1st effectiveness			O		O		O	O	
2nd effectiveness			O		O		O	O	

Exploratory validation	O				O	O
Treatment compliance			O		O	
Adverse event monitoring		O	O	O	O	O

BMI: body mass index, CMT: chuna manual therapy, EA: electroacupuncture, ECG: electrocardiogram, PG: pregabalin, wk: week.

Table S4. Checklist for STRICTA (STandards for Reporting Interventions in Clinical Trials of Acupuncture) 2010

Item	Detail	Description
**1. Acupuncture rationale**	1a) Style of acupuncture	Body Acupuncture
	1b) Reasoning for treatment provided, based on historical context, literature sources, and/or consensus methods, with references where appropriate	1) Relevant article<br>2) Consensus
	1c) Extent to which treatment was varied	Standardized treatment
**2. Details of needling**	2a) Number of needle insertions per subject per session (mean and range where relevant)	21
	2b) Names (or location if no standard name) of points used (uni-/bilateral)	Essential acupuncture points<br>1) ST40 + GB34: EA<br>2) EX-LE 10, EX-UE 11<br>3) TE5 + LI 4: EA<br>4) ST36 + KI6: EA<br>5) CV 4 (unilateral)<br>Selection of treatment points by the operator depending on the location of peripheral neuropathy: EX-LE 12, EX-UE 9
	2c) Depth of insertion, based on a specified unit of measurement or on a particular tissue level	Approximately 1 cm
	2d) Response sought (e.g., de, qi, or muscle twitch response)	Absent
	2e) Needle stimulation (e.g., manual, electrical)	Absent
	2f) Needle retention time	20 min
	2g) Needle type (diameter, length, and manufacturer or material)	0.20 × 30 mm stainless steel sterilized acupuncture needles (Dongbang Acupuncture Inc., Korea)
**3. Treatment regimen**	3a) Number of treatment sessions	Electroacupuncture group: 12 sessions in total
	3b) Frequency and duration of treatment sessions	EA group<br>Treatment period A: 3 sessions/week × 2 weeks<br>Treatment period B: 2 sessions/week × 3 weeks
**4. Other components of treatment**	4a) Details of other interventions administered to the acupuncture	Pregabalin: 150 mg/day (75 mg/capsule, 2 times/day)

	group (e.g., moxibustion, cupping, herbs, exercises, lifestyle advice)	
	4b) Setting and context of treatment, including instructions to practitioners, and information and explanations to patients	The acupuncturist is able to have necessary conversations with the patient.
**5.Practitioner background**	5) Description of participating acupuncturists (qualification or profession affiliation, years in acupuncture practice, other relevant experience)	Individuals who have completed formal education in Korean traditional medicine, obtained a license as a Korean medicine doctor, and have more than 5 years of clinical experience practicing acupuncture
**6. Control or comparator interventions**	6a) Rationale for the control or comparator in the context of the research question, with sources that justify this choice	1) Relevant article
	6b) Precise description of the control or comparator. If sham acupuncture or any other type of acupuncture-like control is used, provide details, as for items 1–3, above.	1) Pregabalin: Usual care<br>2) Pregabalin + CMT

CMT: chuna manual therapy, EA: electroacupuncture.

Table S5. Adverse event details

	Adverse event (AE)	Result[a]	SAE[b]	Severity[c]	Causality[d]	Actions[e]
**PG**	Gum inflammation	1	1	1	5	5
	Elbow bruise	1	1	1	5	5
	Nodules on vocal cords	1	1	1	5	5
**PG + EA**	Rash on the left arm	1	1	1	4	2
	Common cold	1	1	1	5	5
	Left shoulder pain	1	1	2	5	5
	Gastroesophageal reflux disease	1	1	1	5	5
	Chronic cystitis	1	1	1	5	5
**PG + CMT**	Chalazion	2	1	1	5	5
	Bruise	1	1	1	1	2
	Shoulder impingement syndrome	2	1	2	5	8
	Dizziness	2	1	2	5	1
	Head cold	1	1	1	5	5
	Other urticaria	1	1	1	5	5

Herniated disc in the lower back	2	3	2	4	1
Hematuria	1	1	1	5	4
Headache	2	1	2	1	5

a 1: resolved, 2: resolving, 3: not resolved, 4: resolved with sequelae, 5: death, 6: unknown.
b 1: Not a serious adverse event, 2: serious adverse event (SAE) resulting in death or life-threatening situation, 3: SAE requiring hospitalization or prolongation of existing hospital-ization, 4: SAE resulting in persistent or significant disability or incapacity, 5: SAE resulting in congenital anomaly or birth defect, 6: SAE resulting in pregnancy or abortion, 7: SAE involving another medically important condition.
c 1: mild, awareness of signs or symptoms, easily tolerated; 2: moderate, discomfort that interferes with normal daily activities; 3: severe or medically significant, inability to perform normal daily activities; 4: life-threatening, immediate risk to life if reaction persists; 5: death, death due to adverse reaction.
d 1: clearly related, 2: probably related, 3: possibly related, 4: not likely to be related, 5: not related, 6: unable to assess.
CMT: chuna manual therapy, EA: electroacupuncture, PG: pregabalin.
e 1: discontinuation of treatment, 2: reduction of dose, 3: increase of dose, 4: no change in treatment dose, 5: concomitant use of medication, 6: non-drug therapy, 7: unknown, 8: not applicable.

Table S6. Comparison of mean differences in NTX subscales of primary and secondary outcomes (FAS analysis).

		Mean difference			
	Visit	PG (n = 25)	PG + EA (n = 26)	PG + CMT (n = 22)	*p*-value
FACT/GOG-Ntx score	V3–V2	-2.51 (-5.46, 0.43)	-3.93 (-6.80, -1.06) *	-4.03 (-7.11, -0.96) *	0.6664
	V4–V2	-4.42 (-7.37, -1.48) ¶, *	-5.49 (-8.36, -2.62) ¶, *	-6.03 (-9.11, -2.96) ¶	0.6946
	V5–V2	-6.00 (-8.94, -3.05) *	-6.01 (-8.89, -3.14) *	-6.43 (-9.50, -3.37) *	0.9682

CMT: chuna manual therapy; EA: electroacupuncture; PG: pregabalin; V: visit; V1: screening; V2: baseline; V3: treatment 2 weeks; V4: treatment 5 weeks; V5: post follow-up. ¶: primary outcome; *: significant difference within group analysis from baseline as $p < 0.05$. Least squares mean difference and p-values were analyzed by mixed model for repeated measures (MMRM) with the baseline scores, groups, cancer types, and visits as fixed factors, including group*visit and base-line*visit (satisfying normality and homoscedasticity).
